# Draft Genome Sequence of the Syntrophic Lactate-Degrading Bacterium *Tepidanaerobacter syntrophicus* JL^T^

**DOI:** 10.1128/genomeA.01712-15

**Published:** 2016-02-11

**Authors:** Norihisa Matsuura, Akiko Ohashi, Dieter M. Tourlousse, Yuji Sekiguchi

**Affiliations:** Biomedical Research Institute, National Institute of Advanced Industrial Science and Technology (AIST), Tsukuba, Ibaraki, Japan

## Abstract

We report here a high-quality draft genome sequence of the type strain (JL) of *Tepidanaerobacter syntrophicus*, an obligately anaerobic and moderately thermophilic bacterium, which is able to perform syntrophic lactate degradation with hydrogenotrophic methanogens. The genome comprises 2.43 Mb in 9 scaffolds, with a G+C content of 38.6%.

## GENOME ANNOUNCEMENT

*Tepidanaerobacter syntrophicus* is a moderately thermophilic anaerobic bacterium capable of degrading primary aliphatic alcohols and lactate in syntrophic association with hydrogenotrophic methanogens (1). The bacterium was originally isolated from sludge in a full-scale thermophilic (55°C) anaerobic digester decomposing municipal solid wastes, and it was described as a new species of a new genus in the class *Clostridia* of the phylum *Firmicutes*. As a closely related species, *Tepidanaerobacter acetatoxydans* was also isolated and characterized (2), and its genome content was reported (3–5). One of the characteristic differences between the two species lies in the range of substrates utilized, particularly in syntrophic coculture with hydrogenotrophs. While *T. acetatoxydans* can perform anaerobic syntrophic acetate oxidation, such a capability was not observed for *T. syntrophicus* (1). Comparative genomics of the two species may allow us to infer potential genetic features underlying these phenotypic differences related to syntrophy. With this aim, the genome of the type strain of *T. syntrophicus* (strain JL^T^ JCM 12098^T^, NBRC 100060^T^, DSM 15584^T^) was sequenced.

The draft genome of *T. syntrophicus* JL^T^ was generated with a paired-end Nextera XT sequencing library (400- to 600-bp inserts) and a Nextera mate-pair library (1- to 14-kb inserts). The libraries were sequenced on an Illumina NextSeq instrument generating 2 × 150-bp paired-end reads (200× and 50× coverages for the paired-end and mate-pair libraries, respectively). The raw reads were quality trimmed and filtered using Trimmomatic version 0.32 (6). Trimmed reads from the paired-end library were merged with FLASH version 1.2.11 (7), and those from the mate-pair library were processed with NextClip version 1.3.1 (8). For the mate-pair library, the resulting reads in categories A, B, and C were used. Assembly was performed using SPAdes version 3.6.0 (9), followed by additional scaffolding and manual refinement of the assembly, as described previously (10). Annotation of the genome was performed within the Integrated Microbial Genomes (IMG) platform (11).

The final high-quality draft assembly of *T. syntrophicus* JL^T^ contained 10 contigs in 9 scaffolds, for a total length of 2,427,925 bp. The genome had a G+C content of 38.6% and was predicted to contain 2,301 protein-coding sequences and 76 RNA genes. Four complete sets of rRNA genes were identified. Although motility was not observed in our previous study (1), genome annotation indicated the presence of a nearly complete set of genes encoding flagellar proteins, as well as genes for methyl-accepting chemotaxis proteins, suggesting that strain JL^T^ may be motile under certain conditions. The average amino acid identity (AAI) between strain JL^T^ and *T. acetatoxydans* (accession no. NC_019954.2) was estimated to be 77.9% using CompareM (version 0.0.5; GitHub); the two strains shared 1,576 orthologous genes. Further detailed analysis, including genome-scale metabolic modeling, will contribute to our understanding of the physiology of *T. syntrophicus*, including its syntrophic properties*.*

### Nucleotide sequence accession numbers.

The whole-genome shotgun project has been deposited at DDBJ/EMBL/GenBank under the accession number BCMU00000000 (BioProject no. PRJDB4393). The version described in this paper is version BCMU01000000.
